# The Co-Morbidity Burden of Children and Young Adults with Autism Spectrum Disorders

**DOI:** 10.1371/journal.pone.0033224

**Published:** 2012-04-12

**Authors:** Isaac S. Kohane, Andrew McMurry, Griffin Weber, Douglas MacFadden, Leonard Rappaport, Louis Kunkel, Jonathan Bickel, Nich Wattanasin, Sarah Spence, Shawn Murphy, Susanne Churchill

**Affiliations:** 1 Center for Biomedical Informatics, Harvard Medical School, Boston, Massachusetts, United States of America; 2 Children's Hospital Informatics Program, Children's Hospital, Boston, Massachusetts, United States of America; 3 i2b2 National Center for Biomedical Computing, Brigham and Women's Hospital, Boston, Massachusetts, United States of America; 4 Beth Israel Deaconess Medical Center, Harvard Medical School Information Technology, Boston, Massachusetts, United States of America; 5 Center for Developmental Medicine, Children's Hospital, Boston, Massachusetts, United States of America; 6 Program in Genomics, Children's Hospital, Boston, Massachusetts, United States of America; 7 Information Systems Department, Children's Hospital, Boston, Massachusetts, United States of America; 8 Partners Healthcare System Information Technology, Boston, Massachusetts, United States of America; 9 Department of Neurology, Children's Hospital, Boston, Massachusetts, United States of America; 10 Massachusetts General Hospital, Boston, Massachusetts, United States of America; University of Illinois-Chicago, United States of America

## Abstract

**Objectives:**

Use electronic health records Autism Spectrum Disorder (ASD) to assess the comorbidity burden of ASD in children and young adults.

**Study Design:**

A retrospective prevalence study was performed using a distributed query system across three general hospitals and one pediatric hospital. Over 14,000 individuals under age 35 with ASD were characterized by their co-morbidities and conversely, the prevalence of ASD within these comorbidities was measured. The comorbidity prevalence of the younger (Age<18 years) and older (Age 18–34 years) individuals with ASD was compared.

**Results:**

19.44% of ASD patients had epilepsy as compared to 2.19% in the overall hospital population (95% confidence interval for difference in percentages 13.58–14.69%), 2.43% of ASD with schizophrenia vs. 0.24% in the hospital population (95% CI 1.89–2.39%), inflammatory bowel disease (IBD) 0.83% vs. 0.54% (95% CI 0.13–0.43%), bowel disorders (without IBD) 11.74% vs. 4.5% (95% CI 5.72–6.68%), CNS/cranial anomalies 12.45% vs. 1.19% (95% CI 9.41–10.38%), diabetes mellitus type I (DM1) 0.79% vs. 0.34% (95% CI 0.3–0.6%), muscular dystrophy 0.47% vs 0.05% (95% CI 0.26–0.49%), sleep disorders 1.12% vs. 0.14% (95% CI 0.79–1.14%). Autoimmune disorders (excluding DM1 and IBD) were not significantly different at 0.67% vs. 0.68% (95% CI −0.14-0.13%). Three of the studied comorbidities increased significantly when comparing ages 0–17 vs 18–34 with p<0.001: Schizophrenia (1.43% vs. 8.76%), diabetes mellitus type I (0.67% vs. 2.08%), IBD (0.68% vs. 1.99%) whereas sleeping disorders, bowel disorders (without IBD) and epilepsy did not change significantly.

**Conclusions:**

The comorbidities of ASD encompass disease states that are significantly overrepresented in ASD with respect to even the patient populations of tertiary health centers. This burden of comorbidities goes well beyond those routinely managed in developmental medicine centers and requires broad multidisciplinary management that payors and providers will have to plan for.

## Introduction

Over the course of the last 30 years populations of children with autism spectrum disorders have been studied to determine what other non-psychiatric or neuro-cognitive clinical correlates or co-morbidities might exist in this population [1], [2], [3], [4], [5], [6], [7], [8], [9], [10]. Most of these studies have numbered fewer than 200 patients, thus the estimation of prevalence for common co-morbid symptoms and diseases were not accurate. Moreover low-prevalence conditions could not be addressed at all. Nonetheless, given the large numbers of children, possibly as many as 1% [11], with ASD, the need for timely, comprehensive and accurate estimates of these co-morbidities are all the more pressing. This is particularly relevant as we enter an era of accountable care, where baseline data for the cost of each segment of the patient population will be obtained [12]. We have sought to leverage recent developments in informatics technologies for distributed queries across the electronic medical records (EMR) systems data of multiple health care systems to provide a snapshot of this co-morbidity landscape across four hospitals in the Boston area, one a pediatric hospital and three general hospitals with significant pediatric services. In reporting on this study our motivation is first to improve the quantification of the prior estimates of co-morbid conditions such as sleep disorders [13], gastrointestinal complaints and disorders [1], [5], [14], [15], epilepsy [6], cranial anomalies [16], muscular dystrophies [17], [18], [19], [20] and schizophrenia [21] to improve recognition and enable early intervention for these morbidities that further challenge the management of individuals with ASD. Conversely, we also wish to see, for selected co-morbidities previously shown to have a higher prevalence of ASD, what the healthcare system's data estimate that prevalence to be and how they change with age. Given the increased attention that the care of children with ASD has obtained, we hope to stimulate further formal assessment of the symptom and disease association of ASD to allow policy makers and payors to take a full measure of the burden of this common disorder beyond the methods afforded by these EMR-based informatics techniques.

This study includes up to 14,381 individuals (but likely fewer as reviewed in the Discussion) with ASD over a patient population of 2,393,778 below age 35 years (approximately 1 in 200 patients). We employed a query system that does not require a centralized database in order to respect the Health Insurance Portability and Accountability Act (HIPAA), patient consent-to-care, and institutional concerns. This query system entitled Shared Health Resource Information Network (SHRINE) [22] was developed as part of the Harvard Clinical Translational Science award and also leverages the i2b2 National Center for Biomedical Computing [23], [24], [25], [26], [27], [28], [29], [30]

By its very nature, this study can only be exploratory and suggestive. Understanding the degree to which changes in practice or population stratification or referral bias may play in determining the results requires further investigation of the databases and records in each of the individual hospitals which is both beyond the scope of this investigation and indeed is the follow-on activity that tools such as SHRINE are designed to stimulate. Also, this study uses ICD-9 codes (the controlled vocabulary employed principally by healthcare providers to bill for their services), of which some are clearly disease states while others are codes representing symptom complexes. Without chart review we cannot determine if, for example, a diagnosis is determined by symptoms or by diagnostic tests. The ICD-9 codes are also coarse grained and because there are often used for billing, they therefore have potential for bias [31], [32]. Additionally, the assignment of the diagnostic codes will be in part determined by the nature of the specialist seeing the patients which in turn will be driven by what kinds of specialists are available at each hospital [33], [34] Therefore, we expect that the raw prevalence rates may differ from those obtained in prior, smaller, and more fine-grained studies. However, we also expect that there will be a close correspondence in the directionality of increased/decreased co-occurrence of the previously reported co-morbidities and ASD.

Notwithstanding, these limitations, the results presented here summarize one of the largest populations analyzed for the co-morbidity of a common disease that has a growing presence in pediatric clinical practice.

## Methods

### Data Access

We used the Shared Health Research Informatics Network (SHRINE) system [22] for extraction and analysis of whole electronic record datasets from hospital systems equipped with a variety of electronic health care records. This free software has been deployed to over 60 academic health centers internationally. SHRINE in its current implementation only returns aggregate counts of patient populations meeting user-defined criteria based on medications, laboratory data and diagnosis. The Institutional Review Boards of Harvard Medical School, Beth Israel Deaconess Medical Center, Children's Hospital, Boston, and Partners Healthcare Systems have reviewed this system and in the absence of any identifying data and only aggregate counts (i.e. this constitutes non-human subjects research per NIH and institutional guidelines) have approved the research protocol and waived the requirement for consent.

For the purpose of this study we only analyzed diagnoses encoded in the ICD-9 system (see [35]) which is widely used in healthcare and therefore can be used for replication/comparison studies using less data-rich EMR systems available nationally [36]. For example, ASD in this study was defined to include the ICD-9 codes of autistic disorder, Asperger's Syndrome and other pervasive developmental disorders (i.e. ICD 9 codes 299.00, 299.01,299.10,299.11, 299.80, 299.81, 299.90, 299.91—see Table S1). The study covers the period from 2001 to 2010.

### Analysis

All data was analyzed using the base stats package of R. The calculation of chi-square statistics, 95% confidence intervals on the difference in proportions and *p* values were obtained from the prop.test function.

SHRINE queries were composed to determine two sets of fractions or conditional probabilities for those major co-morbidities previously identified for ASD in individuals younger than 35 years of age. We further subcategorized the analyses into age groups 0–17 and 18–34 to see how the co-morbidity prevalence changed across those two age groups.

## Results

**Figure 1:**
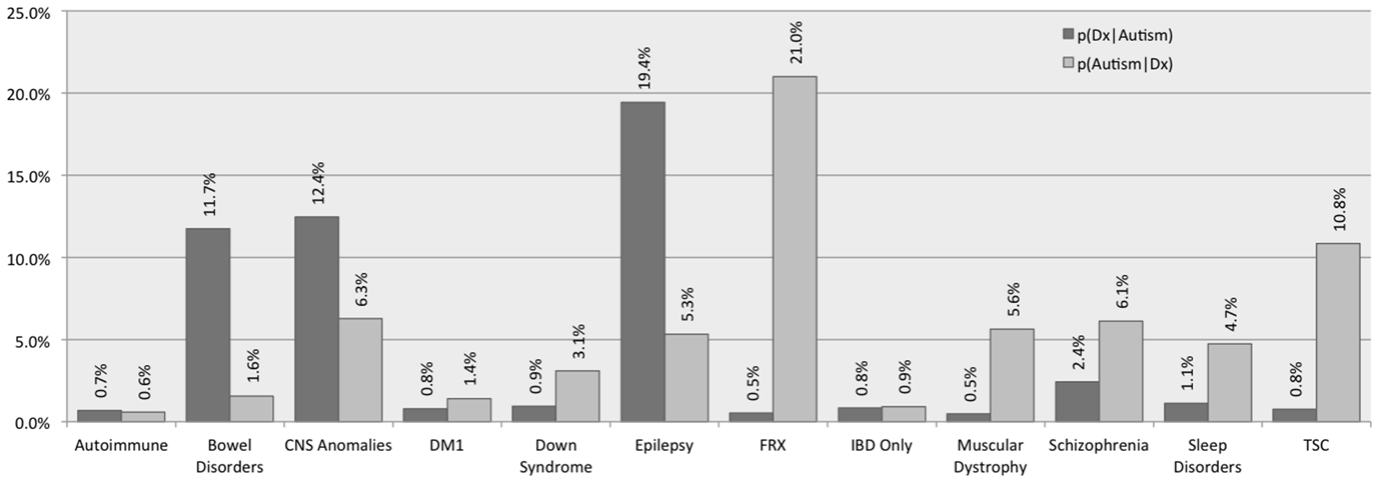
Prevalence of co-morbidities of autism and prevalence of autism in these comorbidities. Shown here is the prevalence of co-morbidities for individuals with autism (denoted as p(Dx}Autism) where Dx is the co-morbidity) and the reciprocal prevalence of autism given the co-morbidity (i.e. p(Autism|Dx)). The prevalence is reported for patients younger than 35 years old. These results are consistent with prior studies and also reinforce that monogenic disorders associated with autism individually only account for a small fraction of the disorder. It also reinforces that autism is present in over 5% of the individuals evaluated for CNS anomalies, epilepsy, muscular dystrophy, schizophrenia, Fragile X Syndrome and Tuberous Sclerosis.

The prevalence of symptoms and co-morbidities with ICD-9 codes corresponding to those diseases previously shown to be co-morbid with ASD are shown in Table 1, and Table S2. Despite the nature of the tools in this study, the estimates obtained are consistent with those from prior studies. Within the group of individuals with ASD, Epilepsy (ICD-9 Diagnosis Code 345.*, see Table S1) was found to have prevalence of 19.4%, schizophrenia 2.4% (83 ICD-9 codes listed in Table S1), bowel disorders 11.7% (112 ICD-9 Codes excluding those for inflammatory bowel disease), inflammatory bowel disease 0.8% (ICD 9 555.* and 556.*), type 1 diabetes mellitus (subset of ICD9 250.* as in Table S1) 0.8%, autoimmune disorders (which for the purpose of this study, specifically excludes IBD and type 1 diabetes mellitus—See Table S1) 0.7%, and CNS/head anomalies (80 ICD-9 codes) 12.4%. Sleep disorders (ICD9 307.4*) at 1.1% were much lower than the prior estimates of 50% obtained in formal assessment of children with ASD [13]. The prevalence of genetic disorders of Fragile X Syndrome, Tuberous Sclerosis and Down Syndrome in individuals with ASD prevalence of 0.5%, 0.8%, 0.9% is consistent with prior studies finding that single gene disorders are responsible for only small fractions of ASD even if collectively they might exceed 20% [37]. Many other genetic syndromes could not be assessed in this study because there was no corresponding ICD-9 code. For other EMR analysis projects we have successfully used natural language processing (NLP) techniques across the clinical notes for these “uncoded” syndromes. Such an approach, which takes considerable time and effort to ensure the accuracy of each phenotype [26], [38], was beyond the scope of this investigation. Also Figure 1 is shown the prevalence of ASD in these same co-morbidities where the aforementioned genetic disorders had ASD prevalences of 21%, 10.8%, and 3.1% respectively. Several other co-morbidities had ASD prevalence of greater than 5% including CNS anomalies (6.3%), epilepsy (5.3%), and schizophrenia (6.1%)

**Table 1:** Characteristics of the ASD population studied.

Characteristic	Pediatric Hospital	General Hospitals	Sum
**Total (All Patients, including ASD)**	1246494	1147284	2393778
**Male, ASD**	7256	4153	11409
**(Female+Male), ASD**	9105	5276	14381
**ASD, Schizophrenia**	125	225	350
**ASD, IBD**	37	82	119
**ASD, Bowel Disorders**	880	808	1688
**ASD, Type 1 DM**	80	34	114
**ASD, Autoimmune Disease**	34	63	97
**ASD, Sleep Disorders**	113	48	161
**ASD, Muscular Dystrophy**	39	29	68
**ASD, Fragile X Syndrome**	47	28	75
**ASD, Tuberous Sclerosis**	31	72	103
**ASD, Epilepsy**	2235	561	2796
**ASD, Down Syndrome**	106	36	142
**ASD, CNS/Cranial anomalies**	900	890	1790

Counts given for patients in the pediatric and general hospital(s), and their sum. All counts are for patients under age 35.

**Figure 2:**
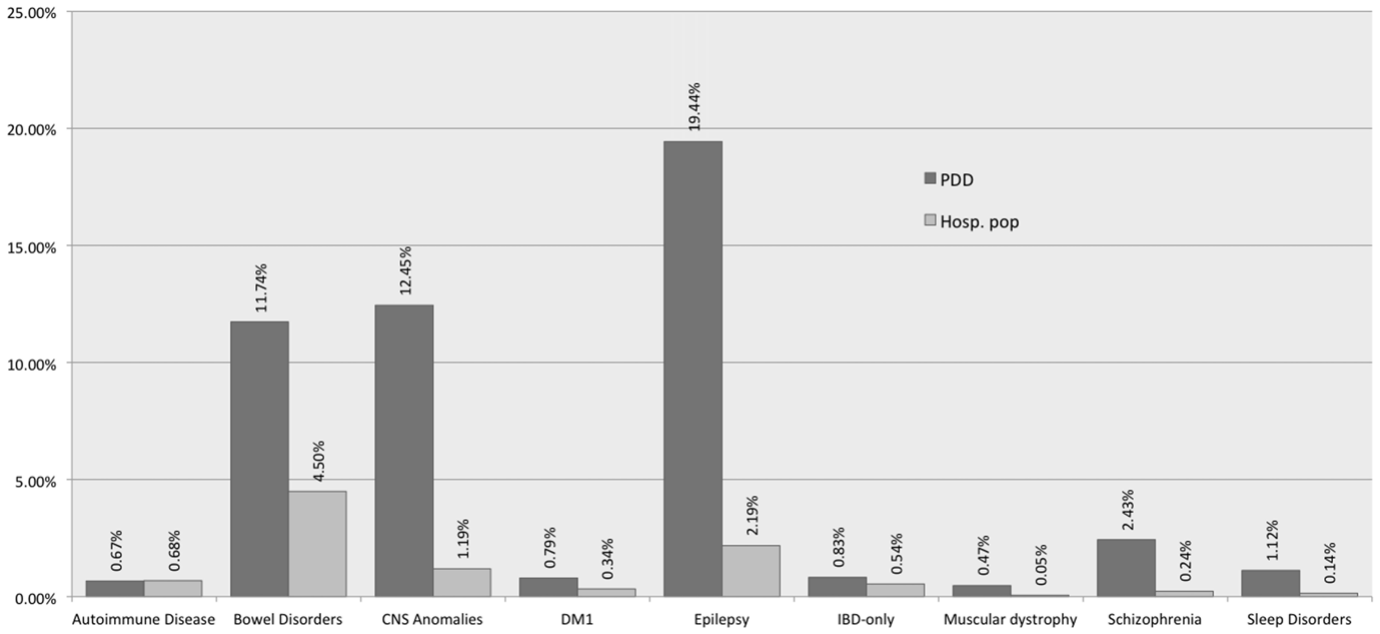
Comorbidities in autism vs same morbidities in the general hospital population. All co-morbities have a significantly different proportion in the ASD population (p<0.0001 by Chi-square) except for the autoimmune diseases (from which IBD and DM1 were excluded and are shown separately on the chart) for which p<0.5. Several of the disorders (epilepsy, muscular dystrophy, schizophrenia, sleep disorders and CNS anomalies have approximately an order of magnitude increased prevalence).

When prevalence of these co-morbidities in ASD were compared to their prevalence in the general population captured by this study (i.e. individuals with at least one inpatient or outpatient visit to one of the four hospitals and therefore enriched for diseases in the population less than 35 years of age—Figure 2), all the above disorders were found to be at significantly higher counts (Pearson's chi-square, p<0.0001, 95% confidence intervals shown in Table 2) except for autoimmune disorders (in ASD at 0.67% vs 0.68%, p<0.97). Even though the category of autoimmune disorders was not significantly greater than the general population, the autoimmune disorders excluded from this category: type 1 diabetes mellitus and IBD were significantly enriched. Also, bowel disorders were found to be significantly increased, a point of some divergence in prior studies [39]. Although the prevalence of sleep disorders in ASD was found to be lower than in prior studies, it nonetheless was approximately ten-fold higher than the general (hospital) population, as was schizophrenia. Muscular dystrophy was almost ten times more prevalent in the ASD population than in the general population, providing quantification absent in prior associations.

**Table 2:** Proportions of morbidity in the subpopulation with ASD and that of the hospital population.

Disorder	Prop in ASD	Prop in hosp. (95^th^ % for Δ)	Prop in ASD, Age 0–17	Prop in hosp. Age 0–17	Prop in ASD, Age 18–34	Prop in hosp. Age 18–34
CNS/cranial anomalies	12.45%	1.19% (9.41–10.38%)	12.56%	2.08%	12.22%	0.44%
Epilepsy	19.44%	2.19% (13.58–14.69%)	19.17%	2.85%	21.45%	1.59%
Schizophrenia	2.43%	0.24% (1.89–2.39%)	1.43%	0.08%	8.76%	0.36%
IBD	0.83%	0.54% (0.13–0.43%)	0.68%	0.21%	1.99%	0.79%
Bowel disorders (not IBD)	11.74%	4.5% (5.72–6.68%)	11.63%	5.02%	12.97%	3.94%
DM1	0.79%	0.34% (0.3–0.6%)	0.67%	0.29%	2.08%	0.37%
Sleep Disorders	1.12%	0.14% (0.79–1.14%)	1.25%	0.2%	0.41%	0.1%
Autoimmune Disorders (not IBD, not DM1)	0.67%	0.68% (−0.14-0.13%)	0.51%	0.23%	0.53%	1.04%
Muscular Dystrophy	0.47%	0.05% 0.26–0.49%	0.49%	0.06%	0.42%	0.04%

Confidence interval shown is the 95^th^ for the difference in the proportions. The columns 2,3 describe the proportions for all ages, columns 4,5 ages 0–17 and columns 6,7 ages 18–34).

**Figure 3:**
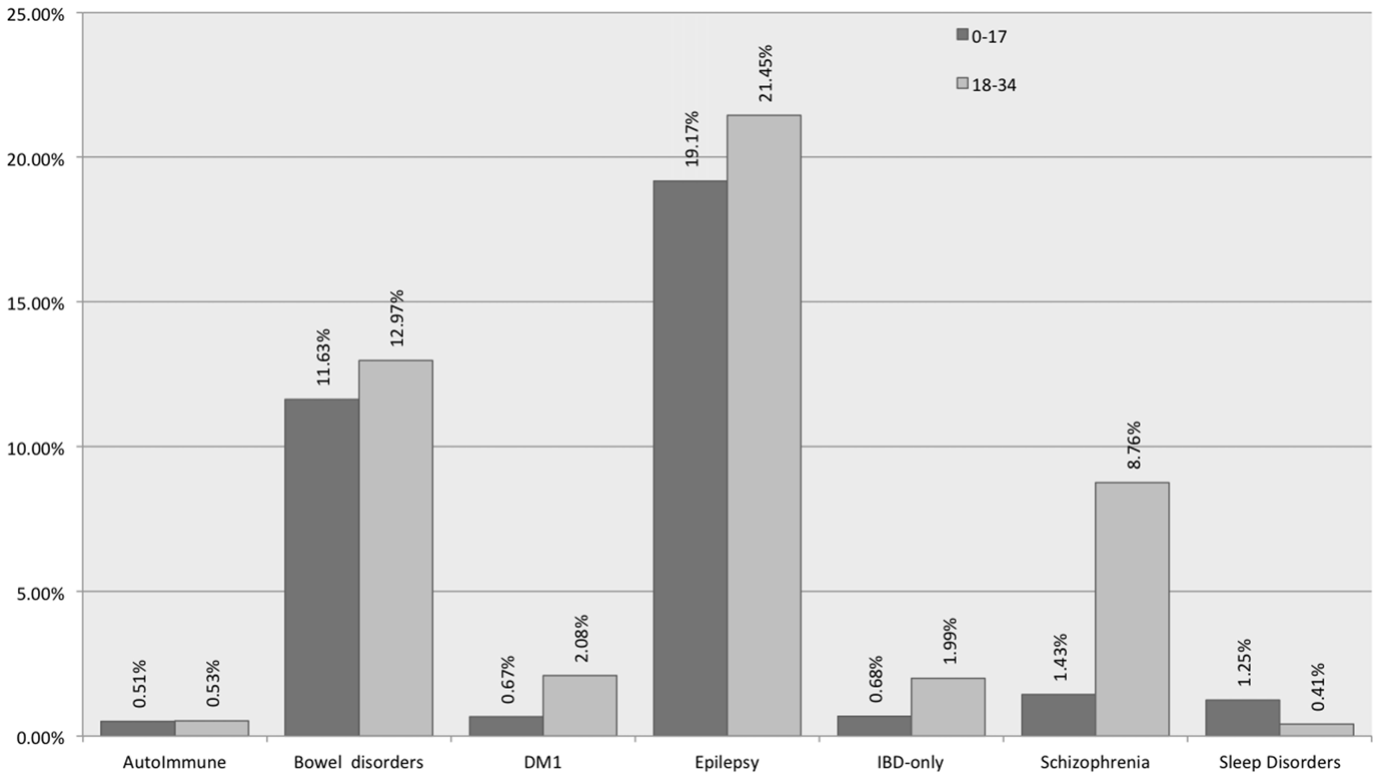
Comorbidities of ASD in younger (0–17 years) vs older (18–34 years). All the comorbidities' prevalence were significantly different (p<0.0001 by Chi square) *except* for bowel disorders, epilepsy, autoimmune disorders (excluding IBD and DM1) and sleep disorders.

A question increasingly asked is how does the comorbidity landscape of ASD change as children become young adults [40], [41]? Consequently we stratified the study population into two age ranges: 0–17 years and 18–34 years. Three co-morbidities did not change significantly with age (using p<0.01 as threshold due to multiple hypothesis testing): bowel disorders (11.63% to 12.97% *p*<0.13), sleep disorders (1.25% to 0.41% p<0.05), and epilepsy (19.17% to 21.45% p<0.05). In contrast DM1 (0.67 to 2.08% p<4.5×10^−10^), IBD (0.68% to1.99% p<9.7×10^−9^) and schizophrenia (1.43% to 8.76%, p<3.1×10^−79^) were significantly different, as in Figure 3. Excluding DM1 and IBD, the other autoimmune disorders did not increase with age.

Although unsurprising, the male to female ratio in this large sample was very close to 4∶1 (see Table 1).

As an additional comparison, we conducted the parallel co-morbidity study for fractures of the lower limb (FxLL) as performed for ASD (see Table S3). All co-morbidities in ASD were significantly higher than those for FxLL with two exceptions: autoimmune disorders (excluding IBD and DM1 but including rheumatological disorders such as rheumatoid arthritis and systemic lupus erythematosus) and DM1. There are several decades of documentation of the higher prevalence of FxLL in both DM1 [42], [43] and the rheumatological diseases (e.g Kim *et al.* [44]).

## Discussion

In this study across multiple health care systems with almost 15,000 patients with ASD, we have been able to corroborate, with a few notable exceptions the co-morbidity findings of prior studies. This study reinforces that burden of co-morbidity is substantial and present across multiple health care systems with over 10 percent of patients with ASD having bowel disorders, or epilepsy, over 5% with CNS or cranial anomalies, and over 2% with schizophrenia. With regard to schizophrenia, although the 2% prevalence was high relative to the general hospital population study, there is a broader controversy of whether or not the population prevalence is 5% or much lower [21], [45].

This study also confirms the high prevalence of ASD in fragile X syndrome, tuberous sclerosis and Down's syndrome. Muscular dystrophy, which mostly is constituted of genetically defined diseases, also has a high prevalence of ASD. The prevalence of ASD in individuals with the diagnosis of epilepsy or schizophrenia in this population is similarly elevated.

Although several studies have previously pointed to an increased prevalence of bowel disorders in individuals with autism/PDD, with up to 25% with three or more gastrointestinal complaints/disorders [1], [5], others have suggested that the prevalence of 9% in ASD is no larger than that of the general pediatric population [39]. In this study, we found a significant enrichment of bowel symptoms and disorders in patients with ASD (11.74% vs. 4.5%, p<0.0001 by chi-square test) but these results may well be affected by the limitations of our study, discussed above. Similarly, sleep disorders have been documented in as many as 25% of all children with ASD [13] but even though the prevalence of sleep disorders in ASD in this study was almost ten times that of the general hospital population, it was nonetheless, only 1.1.%

Of those co-morbidities found to have a higher prevalence than that of the general hospital population studied, three increased significantly with age. Two of them, IBD and DM1 have an autoimmune component to their pathogenesis—a process that large population studies have implicated at least in the risk for ASD, and the third, schizophrenia has been found to have considerable genetic overlap with the ASD population [46], [47], [48], [49].

In addition to the limitations outlined in the introduction, we did not implement a master patient index across the four (often competing) hospitals and therefore some patients may been represented more than once by moving from one hospital to another. Nonetheless, the pediatric hospital alone (ASD N = 9105) and the general hospitals alone (ASD N = 5276), did not have this problem individually and those comorbidities that were found to be significantly enriched across all hospitals were also found in the individual hospitals (data not shown).

These limitations noted above also point to future directions to pursue these investigations. Although it is reassuring that we replicated the approximate co-morbidities of ASD of several diseases found in other studies, the differences with regard to some prior studies do require additional explanation. Short of a prospective study, retrospective studies can be undertaken either with human chart review or by application of natural language processing of the electronically stored clinical notes. Based on our experience in other NLP-driven phenotyping efforts in major depressive disorder, asthma, bipolar disorder, and rheumatoid arthritis [26], [38], this may be both a cost effective and accurate mechanism for a deeper study. The problem of double counting shared patients can and will be addressed by using established mechanisms [50], [51] for automatically matching patients even while maintaining their anonymity.

The differences notwithstanding, this comorbidity topography in four large hospitals presents an informative sketch of the burden of ASD for healthcare system and families alike. It also suggests that this spectrum disorder includes substantial use of specialty clinics outside that of the developmental clinics/center and that this burden increases with age.

## Supporting Information

Table S1 **ICD-9 Codes Used for Disease/Syndrome Definition in Co-morbidity analysis.**
(DOCX)

Table S2 **Hospital patient counts and prevalences for the comorbid conditions across all diagnoses (i.e. not only for ASD) for ages 0–34 years inclusive using the total population of 2,393,778 patients under age 35 to calculate prevalence.**
(DOCX)

Table S3 **Prevalence of ASD Comorbidities for Fractures of the Lower Extremity.** The prevalence is compared to that in ASD and those morbidities with a significant difference (p<0.01) by Chi-square are denoted by *.
(DOCX)

## References

[1] Horvath K, Papadimitriou J, Rabsztyn A, Drachenberg C, Tildon J (1999). Gastrointestinal abnormalities in children with autistic disorder.. The Journal of pediatrics.

[2] Mouridsen SE, Rich B, Isager T (1999). The natural history of somatic morbidity in disintegrative psychosis and infantile autism: a validation study.. Brain Dev.

[3] Mouridsen SE, Rich B, Isager T (1999). Psychiatric morbidity in disintegrative psychosis and infantile autism: A long-term follow-up study.. Psychopathology.

[4] Mouridsen SE, Rich B, Isager T (1999). Epilepsy in disintegrative psychosis and infantile autism: a long-term validation study.. Dev Med Child Neurol.

[5] Horvath K, Perman JA (2002). Autistic disorder and gastrointestinal disease.. Curr Opin Pediatr.

[6] Tuchman R, Rapin I (2002). Epilepsy in autism.. The Lancet Neurology.

[7] Ming X, Brimacombe M, Chaaban J, Zimmerman-Bier B, Wagner GC (2008). Autism spectrum disorders: concurrent clinical disorders.. Journal of Child Neurology.

[8] Bauman ML (2010). Medical comorbidities in autism: challenges to diagnosis and treatment.. Neurotherapeutics.

[9] Charlot L, Abend S, Ravin P, Mastis K, Hunt A (2011). Non-psychiatric health problems among psychiatric inpatients with intellectual disabilities.. J Intellect Disabil Res.

[10] Berlin KS, Lobato DJ, Pinkos B, Cerezo CS, LeLeiko NS (2011). Patterns of medical and developmental comorbidities among children presenting with feeding problems: a latent class analysis.. J Dev Behav Pediatr.

[11] Harrington JW (2010). The actual prevalence of autism: are we there yet?. Pediatrics.

[12] Guterman S, Davis K, Schoenbaum S, Shih A (2009). Using Medicare payment policy to transform the health system: a framework for improving performance.. Health affairs (Project Hope).

[13] Richdale AL, Schreck KA (2009). Sleep problems in autism spectrum disorders: Prevalence, nature, & possible biopsychosocial aetiologies.. Sleep Medicine Reviews.

[14] Ming X, Brimacombe M, Chaaban J (2008). Autism spectrum disorders: concurrent clinical disorders.. Journal of Child Neurology.

[15] Coury D (2010). Medical treatment of autism spectrum disorders.. Curr Opin Neurol.

[16] Smith RD (1981). Abnormal head circumference in learning-disabled children.. Dev Med Child Neurol.

[17] Wu JY, Kuban KCK, Allred E, Shapiro F, Darras BT (2005). Association of Duchenne muscular dystrophy with autism spectrum disorder.. J Child Neurol.

[18] Young HK, Barton BA, Waisbren S, Portales Dale L, Ryan MM (2008). Cognitive and psychological profile of males with Becker muscular dystrophy.. J Child Neurol.

[19] Hendriksen JGM, Vles JSH (2008). Neuropsychiatric disorders in males with duchenne muscular dystrophy: frequency rate of attention-deficit hyperactivity disorder (ADHD), autism spectrum disorder, and obsessive–compulsive disorder.. J Child Neurol.

[20] Hinton VJ, Cyrulnik SE, Fee RJ, Batchelder A, Kiefel JM (2009). Association of autistic spectrum disorders with dystrophinopathies.. Pediatr Neurol.

[21] Morgan CN, Roy M, Chance P (2003). Psychiatric comorbidity and medication use in autism: a community survey.. Psychiatric Bulletin.

[22] Weber GM, Murphy SN, McMurry AJ, Macfadden D, Nigrin DJ (2009). The Shared Health Research Information Network (SHRINE): A prototype federated query tool for clinical data repositories.. J Am Med Inform Assoc.

[23] Murphy SN, Mendis ME, Berkowicz DA, Kohane IS, Chueh HC (2006). Integration of Clinical and Genetic Data in the i2b2 Architecture..

[24] Gainer V, Hackett K, Mendis M, Kuttan R, Pan W (2007). Using the i2b2 hive for clinical discovery: an example..

[25] Brownstein JS, Murphy SN, Goldfine AB, Grant RW, Sordo M (2010). Rapid identification of myocardial infarction risk associated with diabetes medications using electronic medical records.. Diabetes Care.

[26] Liao KP, Cai T, Gainer V, Goryachev S, Zeng-Treitler Q (2010). Electronic medical records for discovery research in rheumatoid arthritis.. Arthritis Care Res (Hoboken).

[27] Murphy SN, Weber G, Mendis M, Gainer V, Chueh HC (2010). Serving the enterprise and beyond with informatics for integrating biology and the bedside (i2b2).. J Am Med Inform Assoc.

[28] Kohane I, Uzuner O (2008). No Structure Before Its Time.. J Am Med Inform Assoc.

[29] Uzuner O, Goldstein I, Luo Y, Kohane I (2008). Identifying patient smoking status from medical discharge records.. Journal of the American Medical Informatics Association: JAMIA.

[30] Uzuner O, Luo Y, Szolovits P (2007). Evaluating the State-of-the-Art in Automatic De-identification.. J Am Med Inform Assoc.

[31] Campbell PG, Malone J, Yadla S, Chitale R, Nasser R (2011). Comparison of ICD-9-based, retrospective, and prospective assessments of perioperative complications: assessment of accuracy in reporting.. J Neurosurg Spine.

[32] Dismuke CE (2005). Underreporting of computed tomography and magnetic resonance imaging procedures in inpatient claims data.. Med Care.

[33] Gabbay V, Coffey BJ, Babb JS, Meyer L, Wachtel C (2008). Pediatric autoimmune neuropsychiatric disorders associated with streptococcus: comparison of diagnosis and treatment in the community and at a specialty clinic.. Pediatrics.

[34] Boyer GS, Templin DW, Bowler A, Lawrence RC, Everett DF (1997). A comparison of patients with spondyloarthropathy seen in specialty clinics with those identified in a communitywide epidemiologic study. Has the classic case misled us?. Arch Intern Med.

[35] World Health Organization (WHO) (2007). International Statistical Classification of Diseases and Related Health Problems: 10th Revision.

[36] Jha AK, DesRoches CM, Campbell EG, Donelan K, Rao SR (2009). Use of electronic health records in U.S. hospitals.. N Engl J Med.

[37] Abrahams B, Geschwind D (2008). Advances in autism genetics: on the threshold of a new neurobiology.. Nat Rev Genet.

[38] Murphy S, Churchill S, Bry L, Chueh H, Weiss S (2009). Instrumenting the health care enterprise for discovery research in the genomic era.. Genome Res.

[39] Black C, Kaye JA, Jick H (2002). Relation of childhood gastrointestinal disorders to autism: nested case-control study using data from the UK General Practice Research Database.. BMJ.

[40] Brugha TS, McManus S, Bankart J, Scott F, Purdon S (2011). Epidemiology of autism spectrum disorders in adults in the community in England.. Archives of general psychiatry.

[41] Tyler CV, Schramm SC, Karafa M, Tang AS, Jain AK (2011). Chronic disease risks in young adults with autism spectrum disorder: forewarned is forearmed.. American journal on intellectual and developmental disabilities.

[42] Forsen L, Meyer HE, Midthjell K, Edna TH (1999). Diabetes mellitus and the incidence of hip fracture: results from the Nord-Trondelag Health Survey.. Diabetologia.

[43] Janghorbani M, Van Dam RM, Willett WC, Hu FB (2007). Systematic review of type 1 and type 2 diabetes mellitus and risk of fracture.. American journal of epidemiology.

[44] Kim SY, Schneeweiss S, Liu J, Daniel GW, Chang CL (2010). Risk of osteoporotic fracture in a large population-based cohort of patients with rheumatoid arthritis.. Arthritis research & therapy.

[45] Taylor MA, Abrams R (1978). The prevalence of schizophrenia: a reassessment using modern diagnostic criteria.. Am J Psychiatry.

[46] Buizer-Voskamp JE, Franke L, Staal WG, van Daalen E, Kemner C (2009). Systematic genotype-phenotype analysis of autism susceptibility loci implicates additional symptoms to co-occur with autism.. AJP: Lung Cellular and Molecular Physiology.

[47] Ingason A, Rujescu D, Cichon S, Sigurdsson E, Sigmundsson T (2009). Copy number variations of chromosome 16p13.1 region associated with schizophrenia.. Molecular Psychiatry.

[48] Eagleson KL, Gravielle MC, Schlueter McFadyen-Ketchum LJ, Russek SJ, Farb DH (2010). Genetic disruption of the autism spectrum disorder risk gene PLAUR induces GABAA receptor subunit changes.. Neuroscience.

[49] Moreno-De-Luca D, Mulle JG, Kaminsky EB, Sanders SJ, Myers SM (2010). Deletion 17q12 is a recurrent copy number variant that confers high risk of autism and schizophrenia.. American journal of human genetics.

[50] Grannis SJ, Overhage JM, Hui S, McDonald CJ (2003). Analysis of a probabilistic record linkage technique without human review..

[51] Grannis SJ, Overhage JM, McDonald CJ (2002). Analysis of identifier performance using a deterministic linkage algorithm..

