# Supplementary material for: The Co-Morbidity Burden of Children and Young Adults with Autism Spectrum Disorders
Source: PLoS One. 2012 Apr 12;7(4):e33224. doi: 10.1371/journal.pone.0033224 (PMC3325235; doi:10.1371/journal.pone.0033224)
Supplement: Table S1 — ICD-9 Codes Used for Disease/Syndrome Definition in Co-morbidity analysis. (DOCX) [file pone.0033224.s001.docx]

Table S1

| ASD | 299.00,299.01,299.10,299.11,299.80,299.81,299.90,299.91, |
| --- | --- |
| Epilepsy | 345.00,345.01,345.10,345.11,345.2,345.3,345.40,345.41,345.50,345.51,345.60,345.61,345.70,345.71,345.80,345.81,345.90,345.91, |
| Bowel Disorder | 456.1,456.21,530.0,530.10,530.11,530.12,530.19,530.20,530.21,530.3,530.4,530.5,530.6,530.81,530.83,530.84,530.85,530.89,530.9,531.10,531.11,531.30,531.31,531.50,531.51,531.70,531.71,531.90,531.91,533.10,533.11,533.30,533.31,533.50,533.51,533.70,533.71,533.90,533.91,534.10,534.11,534.30,534.31,534.50,534.51,534.70,534.71,534.90,534.91,535.00,535.01,535.10,535.11,535.20,535.21,535.30,535.31,535.40,535.41,535.50,535.51,535.60,535.61,540.0,540.1,540.9,541,542,543.0,543.9,560.0,560.1,560.2,560.30,560.31,560.39,560.81,560.89,560.9,562.00,562.01,562.02,562.03,562.10,562.11,562.12,562.13,564.6,565.0,565.1,566,567.0,567.1,567.21,567.22,567.23,567.29,567.38,567.39,567.81,567.82,567.89,567.9,569.0,569.1,569.2,569.41,569.42,569.43,569.49,569.5,ICD9:V12.71 |
| Schizophrenia | 293.81,293.82,295.00,295.01,295.02,295.03,295.04,295.05,295.10,295.11,295.12,295.13,295.14,295.15,295.20,295.21,295.22,295.23,295.24,295.25,295.30,295.31,295.32,295.33,295.34,295.35,295.40,295.41,295.42,295.43,295.44,295.45,295.50,295.51,295.52,295.53,295.54,295.55,295.60,295.61,295.62,295.63,295.64,295.65,295.70,295.71,295.72,295.73,295.74,295.75,295.80,295.81,295.82,295.83,295.84,295.85,295.90,295.91,295.92,295.93,295.94,295.95,297.0,297.1,297.10,297.2,297.3,297.30,297.8,297.9,297.90,298.0,298.1,298.2,298.3,298.30,298.4,298.8,298.80,298.9,298.90,327.95,327.96 |
| AutoImmune Disorder | 279.4,242.00,242.21,359.0,359.1,359.21,373.34,695.4,710.0,710.1,710.2,710.3,710.4,710.8,710.9,714.0,714.1,714.2,714.30,714.31,714.32,714.33,714.4,714.81,714.89,714.9,720.0,725,729.0 |
| DM1 | 250.01,250.03,250.11,250.13,250.21,250.23,250.31,250.33,250.41,250.43,250.51,250.53,250.61,250.63,250.71,250.73,250.81,250.83,250.91,250.93 |
| Sleep Disorders | 307.40,307.41,307.42,307.43,307.44,307.45,307.46,307.47,307.48,307.49 |
| Muscular Dystrophy | 359.0,359.1,359.21 |
| IBD | 555.0,555.1,555.2,555.9,556.0,556.1,556.2,556.3,556.4,556.5,556.6,556.8,ICD9:556.9 |
| CNS/Cranial anomalies | 655.00,655.03,740.0,740.1,740.2,741.00,741.01,741.02,741.03,741.90,741.91,741.92,741.93,742.0,742.1,742.2,742.3,742.4,742.51,742.53,742.59,742.8,742.9,743.00,743.03,743.06,743.10,743.11,743.12,743.20,743.21,743.22,743.30,743.31,743.32,743.33,743.34,743.35,743.36,743.37,743.39,743.41,743.42,743.43,743.44,743.45,743.46,743.47,743.48,743.49,743.51,743.52,743.53,743.54,743.55,743.56,743.57,743.58,743.59,743.61,743.62,743.63,743.64,743.65,743.66,743.69,743.8,743.9,749.10,749.11,749.12,749.13,749.14,749.20,749.21,749.22,749.23,749.24,749.25,ICD9:756.0 |
