# Supplementary material for: The Co-Morbidity Burden of Children and Young Adults with Autism Spectrum Disorders
Source: PLoS One. 2012 Apr 12;7(4):e33224. doi: 10.1371/journal.pone.0033224 (PMC3325235; doi:10.1371/journal.pone.0033224)
Supplement: Table S2 — Hospital patient counts and prevalences for the comorbid conditions across all diagnoses (i.e. not only for ASD) for ages 0–34 years inclusive using the total population of 2,393,778 patients under age 35 to calculate prevalence. (DOCX) [file pone.0033224.s002.docx]

Supplementary Table S2.

| **Condition** | **Pediatric Hospital** | **General Hospitals** | **Prevalence** |
| --- | --- | --- | --- |
| **Bowel Disorders** | 40551 | 67109 | 4.50% |
| **Inflammatory Bowel Disease** | 2601 | 10339 | 0.54% |
| **Cerebral Palsy** | 6611 | 2549 | 0.38% |
| **Autoimmune Disorders** | 2455 | 13838 | 0.68% |
| **Type 1 Diabetes** | 3631 | 4427 | 0.34% |
| **Down Syndrome** | 2701 | 1865 | 0.19% |
| **Epilepsy** | 30739 | 21600 | 2.19% |
| **Fragile X Syndrome** | 229 | 128 | 0.01% |
| **CNS/cranial malformations** | 20936 | 7550 | 1.19% |
| **Muscular dystrophy** | 795 | 1471 | 0.09% |
| **Schizophrenia** | 1091 | 4609 | 0.24% |
| **Sleep disorders** | 2108 | 1295 | 0.14% |
| **Tuberous sclerosis** | 388 | 618 | 0.04% |
