# Supplementary material for: The Co-Morbidity Burden of Children and Young Adults with Autism Spectrum Disorders
Source: PLoS One. 2012 Apr 12;7(4):e33224. doi: 10.1371/journal.pone.0033224 (PMC3325235; doi:10.1371/journal.pone.0033224)
Supplement: Table S3 — Prevalence of ASD Comorbidities for Fractures of the Lower Extremity. The prevalence is compared to that in ASD and those morbidities with a significant difference (p<0.01) by Chi-square are denoted by *. (DOCX) [file pone.0033224.s003.docx]

Supplementary Table S3:

| Comorbidity (in Years 0-34) | Count of comorbidity in patients with fractures of the lower limb, | Prevalence in fractures of the lower limb (%) | Prevalence in ASD (%) |
| --- | --- | --- | --- |
| Autoimmune (not IBD or DM1) | 381 | 1.33% | 0.67%* |
| CNS or head anomalies | 697 | 2.43% | 12.45%* |
| IBD | 185 | 0.65% | 0.83%* |
| DM1 | 227 | 0.79% | 0.79% |
| Epilepsy | 1242 | 4.34% | 19.44%* |
| Schizophrenia | 212 | 0.74% | 2.43%* |
| Sleeping Disorders | 71 | 0.25% | 1.12%* |
| Muscular Dystrophy | 79 | 0.28% | 0.47%* |
| Bowel Disorders | 1165 | 4.07% | 11.47%* |
